# The CacyBP/SIP Protein is Sumoylated in Neuroblastoma NB2a Cells

**DOI:** 10.1007/s11064-013-1155-4

**Published:** 2013-09-28

**Authors:** Urszula Wasik, Anna Filipek

**Affiliations:** Nencki Institute of Experimental Biology, 3 Pasteur Street, 02-093 Warsaw, Poland

**Keywords:** CacyBP/SIP, Neuroblastoma NB2a cells, Post-translational modification, SUMO1

## Abstract

The Calcyclin binding protein and Siah-1 interacting protein (CacyBP/SIP) protein is highly expressed in mammalian brain as well as in neuroblastoma NB2a cells and pheochromocytoma PC12 cells. This protein interacts with several targets such as cytoskeletal proteins or ERK1/2 kinase and seems to be involved in many cellular processes. In this work we examined a post-translational modification of CacyBP/SIP which might have an effect on its function. Since theoretical analysis of the amino acid sequence of CacyBP/SIP indicated several lysine residues which could potentially be sumoylated we checked experimentally whether this protein might be modified by SUMO attachment. We have shown that indeed CacyBP/SIP bound the E2 SUMO ligase, Ubc9, in neuroblastoma NB2a cell extract and was sumoylated in these cells. By fractionation of NB2a cell extract we have found that, contrary to the majority of SUMO-modified proteins, sumoylated CacyBP/SIP is present in the cytoplasmic and not in the nuclear fraction. We have also established that lysine 16 is the residue which undergoes sumoylation in the CacyBP/SIP protein.

## Introduction

The CacyBP/SIP protein (S100A6 binding protein and Siah-1 interacting protein) was originally discovered in Ehrlich ascites tumor cells as an S100A6 binding protein (reviewed in [1]). Later, CacyBP/SIP was found in different mouse, rat and human tissues. A particularly high level of CacyBP/SIP was shown in brain [2, 3] as well as in neuroblastoma NB2a and pheochromocytoma PC12 cells. The immunofluorescence studies have indicated that CacyBP/SIP is present mainly in the cytoplasm and, under certain conditions, also in the nucleus [4, 5]. CacyBP/SIP has several interacting partners. Among them are members of the S100 protein family, for example S100A6 [6], cytoskeletal proteins such as tubulin, actin, tropomyosin as well as ERK1/2 kinase [7–10]. The interaction between CacyBP/SIP and ERK1/2 inhibited phosphorylation of the Elk-1 transcription factor in the nuclear fraction of neuroblastoma NB2a cells and, interestingly, this effect was due to the phosphatase activity of CacyBP/SIP toward ERK1/2 [11].

Sumoylation is a post-translational modification in which the SUMO (Small Ubiquitin Related Modifier) molecule is attached to the target protein. Sumoylation belongs to the same group of modifications as ubiquitination or neddylation. There are four SUMO proteins, SUMO1, SUMO2, SUMO3 and SUMO4. Attachment of SUMO usually occurs on a lysine residue of the target protein located in the consensus sequence ψKxD/E, where “ψ” represents a hydrophobic residue and “x”—any sort of amino acid residue. However, sumoylation might also occur at lysine residues outside the consensus motif. This reaction is catalyzed by three groups of enzymes: E1-activating enzyme, E2-conjugating enzyme and E3-ligase. Up to now, several E3 ligases have been identified and only one E2-conjugating enzyme, namely Ubc9. Sumoylation was shown to be important for many cellular processes such as cell cycle regulation, transcription, protein degradation or chromatin organization [12–14].

Since the theoretical analysis indicated that several lysine residues in the amino acid sequence of CacyBP/SIP might be modified by the SUMO moiety, in this work, using different techniques, we checked the hypothesis that this protein might be sumoylated in neuroblastoma NB2a cells and then we identified the sumoylated amino acid residue in CacyBP/SIP.

## Materials and Methods

### Cell Culture

Mouse neuroblastoma NB2a cells were grown in MEM containing 2 mM l-glutamine, 1 mM sodium pyruvate, 25 mM sodium bicarbonate, 10 % fetal bovine serum, 0.1 mM non-essential amino acids, 100 U/ml penicillin, 100 μg/ml streptomycin and 0.25 μg/ml fungizone. The medium was changed every 2–3 days and cells were passaged when confluent.

### Plasmids

Plasmid pGW1-Ubc9-HA was prepared as follows. The coding region of the Ubc9 protein (E2 SUMO ligase) was obtained by PCR reaction, using cDNA from neuroblastoma NB2a cells as a template and the appropriate primers: forward 5′-TATAGATCTAATGTCGGGGAT-3′ and reverse 5′-TATGAATTCTTATGAGGGCGC-3′. The PCR product was purified from the agarose gel and digested with EcoRI and BglII. Then it was introduced into the pGW1-HA plasmid (gift from Prof. J. Jaworski, International Institute of Molecular and Cell Biology, Warsaw) previously digested with the same enzymes. After transformation into *Escherichia coli* (TOP10F′) and purification, the correctness of the sequence of the cloned insert was confirmed by DNA sequencing.

Plasmids encoding FLAG-tagged CacyBP/SIP with various lysine residues mutated to arginine were generated employing site-directed mutagenesis using pCMV3xFLAG-CacyBP/SIP as a template [7]. The reaction was performed using *Pfu* polymerase and primers listed in Table 1. To remove the parental DNA template the PCR product was treated with *DpnI* endonuclease. The introduced mutations were verified by DNA sequencing.

**Table 1:** Sequences of primers used for K/R mutagenesis in CacyBP/SIP

Residue	Primers
K16R	Fw: CCTAGAAGAGGTCAGAGTATTGCTGGAAAAG
	Rv: GACTTTTCCAGCAATACTCTGACCTCTTCTA
K37R	Fw: GTGAAAAGTCCAGGATTGAGACGGAAC
	Rv: GAGTTCCGTCTCAATCCTGGACTTTTC
K43R	Fw: GATTGAGACGGAACTCCGGAACAAGATGCAAC
	Rv: GTTGCATCTTGTTCCGGAGTTCCGTCTCAATC
K49R	Fw: GATGCAACAGCGGTCGCAGAAGAAA
	Rv: GGTTTCTTCTGCGACCGCTGTTGCA
K52R	Fw: TGCAACAGAAGTCGCAGCGGAAACCAGAACTTGAT
	Rv: ATCAAGTTCTGGTTTCCGCTGCGACTTCTGTTGCA
K53R	Fw: GAAGTCGCAGAAGCGACCAGAACTGATAA
	Rv: CATTATCAAGTTCTGGTCGCTTCTGCGACT
K147R	Fw: GTTCAAAAAAAGTCCGGACT GATACAGTA
	Rv: ACTGTATCAGTCCGGACTTTTTTTGAACT
K208R	Fw: CGGAGACGATGATATGAGG CGAACCATTAA
	Rv: TATTAATGGTTCGCCTCATATCATCGTCTC

Plasmids pCMV-FLAG-Ubc9 encoding FLAG-tagged Ubc9 and pEYFP-SUMO1 encoding SUMO moiety fused with EYFP were a generous gift from Prof. J. M. Henley (Department of Biochemistry, School of Medical Sciences, University of Bristol, UK).

### Cell Transfection and Co-precipitation of CacyBP/SIP with Ubc9

To check if Ubc9 co-precipitates with CacyBP/SIP in the cell extract, neuroblastoma NB2a cells were co-transfected with plasmids encoding Ubc9-HA (pGW1-HA-Ubc9) and CacyBP/SIP-FLAG (pCMV3xFLAG-CacyBP/SIP) or, as a control, encoding Ubc9-HA only (pGW1-HA-Ubc9 and pCMV3xFLAG) using Lipofectamine 2000 (Invitrogen). After 24 h cells were harvested and suspended in IP buffer containing 50 mM Tris–HCl (pH 7.5), 50 mM KCl, 1 mM EDTA, 1 mM MgCl_2_, 0.1 % Triton X-100, protease inhibitor mixture (Complete, Roche Molecular Biochemicals) and 20 mM *N*-ethylmaleimide (NEM). Next, the extract was centrifuged for 10 min at 16,000×g. Proteins from the supernatant fraction (800 μg) were incubated with 40 μl of anti-FLAG M2 agarose resin (Sigma), previously equilibrated with IP buffer, for 3 h at 4 °C. Unbound proteins were removed, the resin was washed five times with IP buffer and bound proteins were eluted from the anti-FLAG agarose resin using 0.1 M glycine–HCl, pH 3.5. Proteins were then separated on 10 % SDS gel and identified by Western blot using the appropriate antibodies.

### Sumoylation of CacyBP/SIP in Neuroblastoma NB2a Cells

To check the sumoylation of CacyBP/SIP, neuroblastoma NB2a cells were co-transfected with plasmids: pEYFP-SUMO1, pCMV-FLAG-Ubc9 and pCMV3xFLAG-CacyBP/SIP. The cells were subjected to fractionation in order to obtain cytoplasmic and nuclear fractions using NE-PER extraction reagents (Pierce) according to the manufacturer’s instruction (purity of the cytoplasmic and nuclear fraction was checked using anti-GAPDH or anti-HDAC1 antibodies, respectively) or suspended directly in the RIPA buffer containing: 10 mM Tris–HCl (pH 7.5), 150 mM NaCl, 1 mM EDTA, 1 mM EGTA, 0.5 % NP-40, 1 % Triton X-100. Proteins from the cell extract (80 μg) were analyzed by Western blot using monoclonal antibodies against CacyBP/SIP (Abcam).

### SDS-PAGE and Western Blotting

Gel electrophoresis with 10 % (w/v) polyacrylamide containing 0.1 % SDS was performed by the method of Laemmli [15]. Proteins were transferred electrophoretically onto nitrocellulose and identified using primary antibodies: mouse anti-CacyBP/SIP monoclonal antibody (1:1,000; Abcam), or mouse anti-Ubc9 antibody (1:1,000; Sigma). After washing with TBS-T buffer (50 mM Tris, pH 7.5, 200 mM NaCl, 0.05 % Tween 20) the blots were incubated with secondary goat anti-mouse IgG antibodies conjugated to horseradish peroxidase (1:10,000) (Jackson Immunoresearch Laboratories). After three washes with TBS-T and two washes with TBS (50 mM Tris, pH 7.5, 200 mM NaCl) blots were developed with the ECL chemiluminescence kit (Amersham Biosciences) followed by exposure against an X-ray film.

## Results

### CacyBP/SIP Co-precipitates with Ubc9 in Neuroblastoma NB2a Cells

**Fig. 1:**
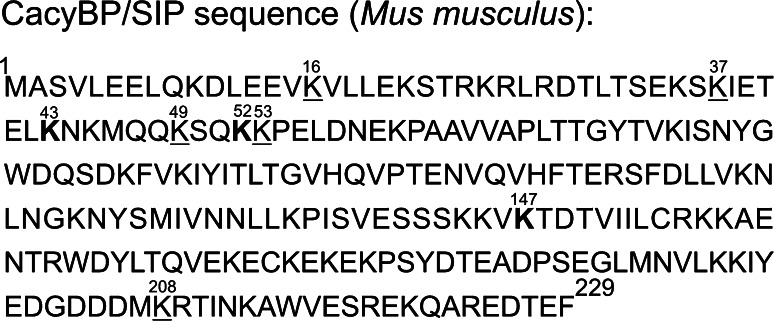
The amino acid sequence of mouse CacyBP/SIP (accession number NP_033916). Lysine residues, identified by SUMOsp 2.0 tool, which might be potentially sumoylated are marked in bold (these with high probability) or underlined (these with lower probability)

The CacyBP/SIP amino acid sequence was analyzed by the SUMOsp 2.0 online tool (http://sumosp.biocuckoo.org). As it is shown in Fig. 1, several potential sumoylation sites were identified. Among them were lysine residues in positions 16, 37, 43, 49, 52, 53, 147 and 208.

**Fig. 2:**
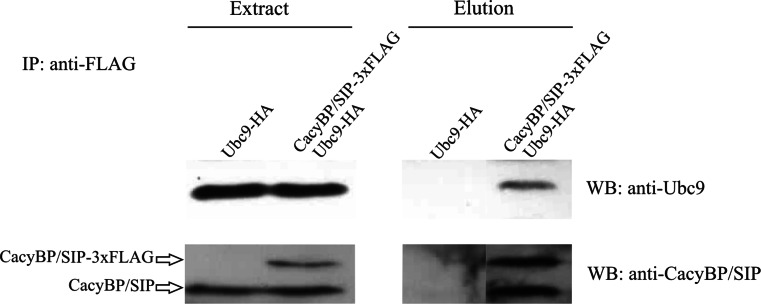
Co-immunoprecipitation of CacyBP/SIP with Ubc9 in NB2a cell extract. Cells were co-transfected with pGW1-HA-Ubc9 and pCMV3xFLAG-CacyBP/SIP (designated as HA-Ubc9 and 3xFLAG-CacyBP/SIP) or pGW1-HA-Ubc9 and pCMV3xFLAG (designated as HA-Ubc9). Proteins from the extract were used directly for Western blot analysis (80 μg) or incubated with anti-FLAG M2 affinity resin (800 μg). Proteins co-precipitated with CacyBP/SIP-3xFLAG were examined by Western blot using anti-Ubc9 or anti-CacyBP/SIP antibodies. Western blot images representative for 3 experiments performed are shown

In order to establish whether CacyBP/SIP might be sumoylated we first checked if it bound the SUMO conjugating enzyme, Ubc9. To examine this, a co-immunoprecipitation assay was performed. Ubc9-HA and CacyBP/SIP-3xFLAG were transiently overexpressed in mouse neuroblastoma NB2a cells and proteins from the cell extract were incubated with anti-FLAG M2 affinity resin to isolate CacyBP/SIP-3xFLAG with its targets. Proteins in the elution fraction were examined by Western blot analysis for the presence of Ubc9. As shown in Fig. 2, Ubc9-HA co-precipitated with CacyBP/SIP-3xFLAG, indicating that CacyBP/SIP and Ubc9 interacted with one another and suggesting that CacyBP/SIP might undergo sumoylation.

### CacyBP/SIP is Sumoylated in Neuroblastoma NB2a Cells and is Present in the Cytoplasmic Fraction

**Fig. 3:**
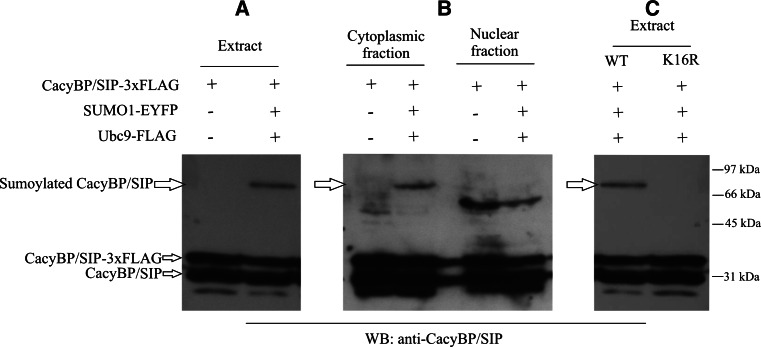
Sumoylation of CacyBP/SIP in neuroblastoma NB2a cells. Western blot developed with anti-CacyBP/SIP antibody shows sumoylated CacyBP/SIP in cell extract (**A**) and in subcellular fractions of NB2a cells (**B**). In each case 80 μg of protein was applied on the gel. Panel **C** shows that wild type CacyBP/SIP (WT) is sumoylated in NB2a cell extract while K16R CacyBP/SIP mutant is not. Western blot images representative for 3 experiments performed are shown

To examine if CacyBP/SIP is sumoylated in the cell, neuroblastoma NB2a cells were transfected with plasmids encoding proteins that enhance this modification i.e., Ubc9 and SUMO as described in Materials and Methods. As it is seen in Fig. 3A an additional band recognized by anti-CacyBP/SIP antibody at the level of 75 kDa becomes visible. This band, representing EYFP-SUMO1-CacyBP/SIP-3xFLAG, is detected in cells overexpressing SUMO1-EYFP, Ubc9-FLAG and CacyBP/SIP-3xFLAG but not in cells overexpressing only CacyBP/SIP-3xFLAG. In order to establish the subcellular localization of sumoylated CacyBP/SIP, NB2a cells were subjected to fractionation into nuclear and cytoplasmic fractions. As it can be seen in Fig. 3B, the EYFP-SUMO1-CacyBP/SIP-3xFLAG band migrating at the level of 75 kDa is present in the cytoplasmic fraction and not in the nuclear one. In the nuclear fraction a band migrating at the level of 66 kDa was detected with anti-CacyBP/SIP antibody, which most probably represents CacyBP/SIP dimer [16].

### Lysine 16 is an Acceptor of SUMO in CacyBP/SIP

The theoretical analysis performed by the SUMOsp 2.0 online tool allowed us to identify several lysine residues in the CacyBP/SIP amino acid sequence that might undergo sumoylation. To establish which lysine residue is modified by SUMO1, several mutants, in which particular lysine residues were replaced by arginine, were constructed. Interestingly, only the mutant in which lysine in position 16 was changed to arginine was not sumoylated. This observation allowed us to conclude that this particular lysine is a target for SUMO1 attachment (Fig. 3C).

## Discussion

The CacyBP/SIP protein is distributed in various tissues and cells, mainly in brain neurons or in established cell lines of neuronal origin such as neuroblastoma NB2a cells, and seems to play a role in various cellular processes (reviewed in [1]. Recently, we have shown that CacyBP/SIP has phosphatase activity and that this activity depends on CacyBP/SIP phosphorylation [11, 17, 18]. To explore the post-translational modifications of CacyBP/SIP, that might have an impact on its function, in this work we focused on sumoylation. Theoretical analysis indicated several lysine residues in the CacyBP/SIP sequence which might be modified by the SUMO molecule. Indeed, our experimental data confirmed that CacyBP/SIP was sumoylated in neuroblastoma NB2a cells, and moreover, we identified a lysine residue involved in this modification.

Sumoylation was shown to strongly determine the subcellular localization of proteins and to be important in key cellular processes. For a long time this modification was considered to be limited to the nucleus, however, the evidence of cytoplasmic protein sumoylation, especially in the nervous system, has emerged recently [19]. For instance, sumoylation of the cytoplasmic La protein is important for the axonal transport. Sumoylated La protein interacts with dynein, which facilitates the retrograde transport in neurons, whereas its non-sumoylated form interacts with kinesin, which is responsible for the anterograde transport [20]. Sumoylation of cytoskeletal proteins seems to be implicated in different cytoskeletal functions such as dendritic spine formation [21]. Since CacyBP/SIP interacts with tubulin, actin and tropomyosin [7–9], one can speculate that CacyBP/SIP sumoylation may play a role in the organization of the cytoskeleton, especially during cell differentiation.

Recently, it has been reported that sumoylation might have an effect on the enzymatic activity of some proteins. For instance, it was shown, that SUMO attachment regulates the activity of a protein tyrosine phosphatase, PTP1B. Sumoylation of PTP1B inhibits its activity [22] and affects its subcellular localization [23]. Another example is the focal adhesion kinase, FAK. Sumoylation of FAK dramatically increases its autophosphorylation and activity [24]. Regarding CacyBP/SIP, it is known that it exhibits phosphatase activity towards ERK1/2 and tau [11, 18] but we have not seen any effect of CacyBP/SIP sumoylation on dephosphorylation of these substrates (data not shown).

**Fig. 4:**
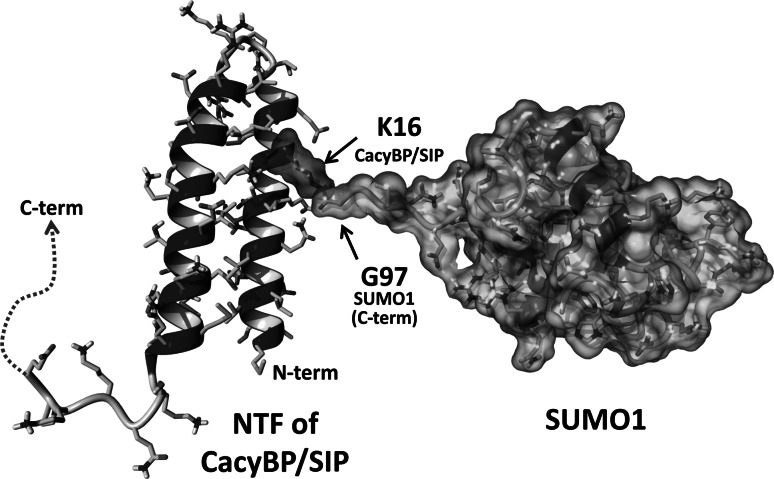
Model of the CacyBP/SIP N-terminal fragment (NTF) with bound SUMO1. The NTF structure of mouse CacyBP/SIP was taken from the Protein Data Bank (PDB; id:1YSM) as the first model of the 20 NMR structures [27]. The structure of human SUMO1, which shares 100 % amino acid sequence identity with the mouse ortholog, was extracted from a crystal structure of the human SUMO E1 complex (PDB; id:3KYC) [28]. Both proteins were bonded via residues K16 from NTF of CacyBP/SIP and G97 from the SUMO1 C-terminus. The K16 residue of CacyBP/SIP (*red*) and SUMO1 molecule are shown in semitransparent surfaces (Color figure online)

To explain the lack of effect of CacyBP/SIP sumoylation on its functions we have analyzed the structure of the modified protein. For that we have employed a molecular modeling method using the available structures of CacyBP/SIP N-terminus and of SUMO1. As it can be seen in Fig. 4, the N-terminal part of CacyBP/SIP forms two anti-parallel helices (47 amino acids). The K16 residue is located on the side of the helix not facing the other helix therefore the attachment of a moiety, even one as large as SUMO1, should not significantly affect the structure of CacyBP/SIP. The exposed location of the SUMO moiety on CacyBP/SIP makes it easily accessible for other proteins. Interestingly, sumoylation, similarly to some other posttranslational modifications, for instance acetylation, is regarded as a modification that may help to assemble large protein complexes since the SUMO moiety can be recognized by a SUMO interacting motif (SIM) present in other proteins [25]. Thus, CacyBP/SIP sumoylation may facilitate its interaction with other protein partners. This feature is important since CacyBP/SIP was postulated to be a component of multiprotein complexes [26].

Taken together, the CacyBP/SIP protein is involved in many cellular processes under normal and pathological conditions but little is known about the mechanisms which determine its activity. CacyBP/SIP sumoylation might be considered as an important modification that might facilitate the formation of multiprotein complexes involved in the organization of the cytoskeleton, especially during differentiation of neuronal type of cells.

## Abbreviations

CacyBP/SIP: Calcyclin binding protein and Siah-1 interacting protein
PAGE: Polyacrylamide gel electrophoresis
SDS: Sodium dodecyl sulfate
SUMO: Small ubiquitin related modifier
Ubc9: SUMO conjugating enzyme
